# Stimulation in the Rat Anterior Insula and Anterior Cingulate During an Effortful Weightlifting Task

**DOI:** 10.3389/fnins.2021.643384

**Published:** 2021-02-26

**Authors:** Carlos Silva, Blake S. Porter, Kristin L. Hillman

**Affiliations:** Department of Psychology, Brain Health Research Centre, University of Otago, Dunedin, New Zealand

**Keywords:** effort, insula, cingulate cortex, persistence, rat, weightlifting

## Abstract

When performing tasks, animals must continually assess how much effort is being expended, and gage this against ever-changing physiological states. As effort costs mount, persisting in the task may be unwise. The anterior cingulate cortex (ACC) and the anterior insular cortex are implicated in this process of cost-benefit decision-making, yet their precise contributions toward driving effortful persistence are not well understood. Here we investigated whether electrical stimulation of the ACC or insular cortex would alter effortful persistence in a novel weightlifting task (WLT). In the WLT an animal is challenged to pull a rope 30 cm to trigger food reward dispensing. To make the action increasingly effortful, 45 g of weight is progressively added to the rope after every 10 successful pulls. The animal can quit the task at any point – with the rope weight at the time of quitting taken as the “break weight.” Ten male Sprague-Dawley rats were implanted with stimulating electrodes in either the ACC [cingulate cortex area 1 (Cg1) in rodent] or anterior insula and then assessed in the WLT during stimulation. Low-frequency (10 Hz), high-frequency (130 Hz), and sham stimulations were performed. We predicted that low-frequency stimulation (LFS) of Cg1 in particular would increase persistence in the WLT. Contrary to our predictions, LFS of Cg1 resulted in shorter session duration, lower break weights, and fewer attempts on the break weight. High-frequency stimulation of Cg1 led to an increase in time spent off-task. LFS of the anterior insula was associated with a marginal increase in attempts on the break weight. Taken together our data suggest that stimulation of the rodent Cg1 during an effortful task alters certain aspects of effortful behavior, while insula stimulation has little effect.

## Introduction

The ability to appropriately persevere or abandon effortful tasks is essential for optimal function (Hull, 1943). Persisting through effort is often needed to achieve highly valued rewards, but organisms must also know when to quit behaviors or tasks that are no longer optimal based on external and/or internal signals (Stephens and Krebs, 1986; Hockey, 2013). Behavioral disruptions in either direction are observed in certain human pathologies, including attention deficit hyperactivity disorder, obsessive-compulsive disorder, and depressive disorders (American Psychiatric Association, 2013; Chong et al., 2016; Pessiglione et al., 2018).

Neural activity during the acute decision phase of selecting a high-effort, high-reward course of action has been studied in multiple species, including humans (Croxson et al., 2009; Engstrom et al., 2014; Arulpragasam et al., 2018), laboratory rats (Bardgett et al., 2009; Ostrander et al., 2011; Cowen et al., 2012), macaque monkeys (San-Galli et al., 2018), and marmosets (Enomoto et al., 2018). However, there are far fewer studies examining neural activity *after* the initial decision phase, i.e., what drives an animal to continue to persist in (or quit) an effortful task once the task has been initiated? Neurocognitive frameworks of fatigue suggest that extended effort expenditure recruits functional connectivity between the anterior cingulate cortex (ACC), the anterior insula, and the lateral prefrontal cortex (Muller and Apps, 2019). It is not known though whether modifying activity in any of these regions can alter persistence (or quitting) behaviors in a given task.

The ACC [cingulate cortex area 1 (Cg1) in rodent] is a region with known involvement in motivated behavior. Electrical stimulation of the human ACC at 50 Hz evokes subjective reports of motivation to accomplish goals and surpass challenges (Parvizi et al., 2013). Similarly, ablation of ACC in humans is sufficient to reduce some of the cognitive symptoms of obsessive-compulsive disorder (Sheth et al., 2013). In laboratory rats, neurons in Cg1 encode effort-outcome values (Hillman and Bilkey, 2010, 2012; Cowen et al., 2012) and manipulations of this region affect an animal’s preference for high-effort, high-reward courses of action (Walton et al., 2003; Schweimer and Hauber, 2005, 2006; Rudebeck et al., 2006). Others report, however, that Cg1 activity might mediate some types of effortful action, but not all (Holec et al., 2014). This inconsistency regarding the precise role of the ACC/Cg1 in effort-laden motivated behavior is not surprising given the wide range of phenomena and functions ascribed to the ACC/Cg1, including autonomic regulation, fear and anxiety, nociception, and attention (Medford and Critchley, 2010). ACC/Cg1’s involvement in diverse functions suggests it is a major node in high-order cognitive control circuitry, including that required for complex, dynamic decision making (Heilbronner and Hayden, 2016; Kolling et al., 2016; Wang et al., 2018).

Like the ACC, the anterior insula [broadly homologous to the agranular insular (AI) in rodent] is implicated in a wide range of phenomena, including aggression, fear, interoception, frustration, and food- and drug-seeking behaviors (Craig, 2009). Of note, the AI is involved in mediating behavioral responses to changes in cost-benefit parameters: both lesioning and GABAergic inhibition of AI in rodents promotes the pursuit of higher food outcomes in tasks with reward devaluation schedules (Balleine and Dickinson, 2000; Parkes et al., 2015). These findings have found resonance in similar experiments with cocaine (Moschak et al., 2018) and nicotine (Pushparaj et al., 2013). In human experiments when feelings of frustration are induced by blocking participants’ progression in a task, there is coincident activation of a network that includes the anterior insula (Yu et al., 2014). In another human experiment, self-reported feelings of satisfaction after curiosity-inducing tasks are associated with insular activity (Lee and Reeve, 2017). Taken together, these results suggest that the anterior insula plays a central role in controlling internal motivational states which may influence persistence or quitting behaviors.

The ACC and anterior insula have been proposed to functionally interact as a Salience Network (Medford and Critchley, 2010; Menon and Uddin, 2010). This network – which can facilitate network shifts between the Default Mode Network and Central Executive Network – helps an animal appropriately respond to salient cues, whether those cues stem from challenges and changes in environment, expectations, preferences, and/or internal signals (Medford and Critchley, 2010; Scholl et al., 2015). All of these cue types dynamically change during an effort-laden task, suggesting that Salience Network node activity may be critical in driving – or dissuading – persistence in the task at hand.

Here we used a laboratory rat model to investigate whether electrical stimulations of ACC/Cg1 or AI change an animal’s persistence in an effortful weightlifting task (WLT). We tested a low (10 Hz) and a high (130 Hz) frequency as behavioral effects of stimulation are often influenced by frequency (Mohan et al., 2020). For example in kindled rats, seizure activity can be precipitated with 10 Hz hippocampal stimulation but suppressed with130 Hz (e.g., Wyckhuys et al., 2010). We predicted that 10 Hz Cg1 stimulation would increase persistence in the WLT given that Cg1 activity in rodent is linked to high-effort, high-reward choice behavior, and that mid-frequency (50 Hz) pre-operative ACC stimulation in humans has been associated with a “will to persevere” (Parvizi et al., 2013). We predicted that 130 Hz AI stimulation would reduce task engagement and persistence, given that a previous study linked 130 Hz insular stimulation to reduced nicotine self-administration in a progressive ratio operant task (Pushparaj et al., 2013).

## Materials and Methods

### Animals

Ten male Sprague-Dawley rats (*n* = 10) were sourced from the University of Otago’s Hercus Taieri Resource Unit (Dunedin, New Zealand) and housed in 38 × 30 × 35 cm clear plexiglass, individually ventilated cages (Tecniplast, Italy). At the beginning of experiments, rats were approximately 7 months of age with an average body weight of 439 g (±5.8 g). At the end of experiments, rats were approximately 10 months of age with an average body weight of 436 g (±5.1 g). Animals were paired in cages but kept separate by a clear, perforated barrier so auditory and olfactory interaction could happen, but no direct physical contact could occur. All animals were kept on a 12 h reverse dark-light cycle, with all experimental procedures being conducted during the animal’s dark phase. The rats were kept on a restricted diet of standard rat chow (Teklad diet, Envigo, United States) to limit their body weight and promote interest in food reward; all rats were maintained at ≥85% of their free-feeding body weight. Water was available *ad libtum.* All procedures were approved by the Animal Ethics Committee at the University of Otago, protocol 91/17.

### Weightlifting Task

**FIGURE 1:**
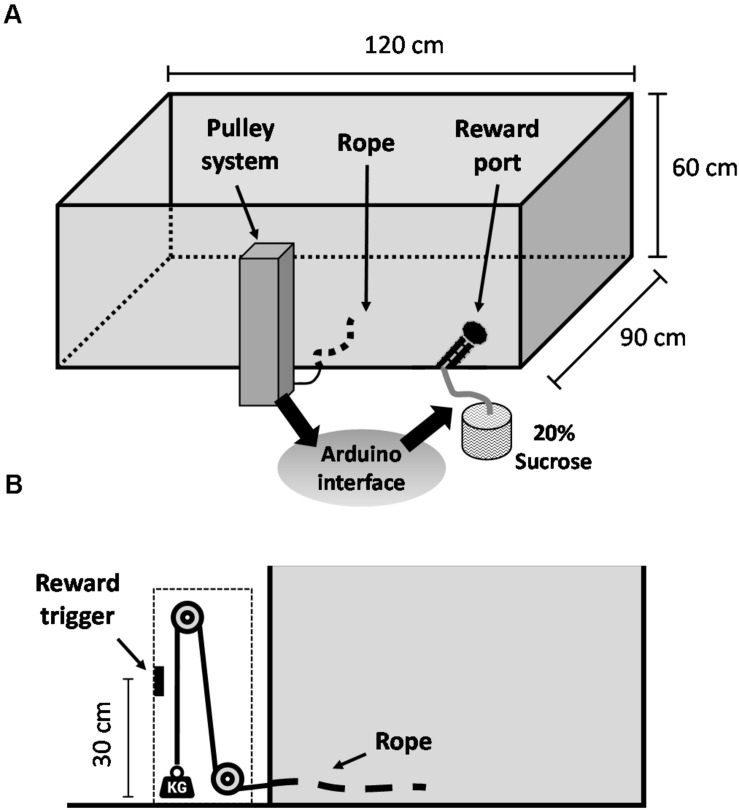
Weightlifting task schematic. **(A)** General overview of the apparatus. **(B)** Profile view of the weighted pulley system that allows for effort manipulations within the task. Video footage of the apparatus and of the task in action is included in the supplementary material of Porter and Hillman (2019).

Before surgery, animals were trained in the WLT, a novel effort expenditure task that was recently developed and validated by our lab. For a detailed description of the WLT apparatus, materials, and full training procedures, see Porter and Hillman (2019); videos of the task in action are included in the supplementary material of that publication. In brief, the WLT consists of a 120 cm × 90 cm × 60 cm wooden open arena, painted black. Inside the arena, two conduit pipes extend from the wall: one conduit contains a rope, which is connected outside the arena to a vertical pulley system; the other conduit contains a silicone tube, which is connected outside the arena to a peristaltic pump containing 20% sucrose liquid (Figure 1A). The animal must pull the rope 30 cm to trigger automated dispensing of 0.2 ml sucrose reward. The pulley system enables different weights to be added to the rope, ranging 45–225 g in 45 g increments, thus increasing the difficulty of rope pulling within the arena (Figure 1B). Animals were initially trained on a rope containing no weight (“0 g”) and once proficient were challenged with 45 g. Training was considered complete once an animal was able to perform 10 successful pulls of 0 g, immediately followed by 10 successful pulls of 45 g, all within 5 min. The WLT is automated via an Arduino microcontroller, which is configured to send TTL signals to a nearby acquisition system (Digital Lynx SX; Neuralynx Inc.) for timestamping of all task events.

### Surgery

Once trained in the WLT, animals were prepared for surgical implantation of electrodes. Rats were placed in an induction chamber and given 5% isoflurane in oxygen mixture (EZ-7000, EZ Anesthesia, United States). Once voluntary movement ceased, the animals were removed from the chamber and placed in a stereotaxic frame (Stoelting, United States) equipped with non-traumatic ear bars and a nose cone for anesthetic maintenance at 2–4%. The animals received subcutaneous doses of amphoprim (trimethoprim and sulphadimethyl pyrimidine, 30 mg/kg), atropine (0.065 mg/kg), and buprenorphine (0.05 mg/kg). The scalp was infused with subcutaneous bupivacaine (4 mg/kg). A single incision was made over the scalp and the skull exposed, bregma and lambda were located and the two landmarks were leveled via nose bar adjustment. Craniotomies were drilled for electrode implantations and for the placement of structural screws. Coordinates for the implants, taken from bregma, were: AP +3.7, ML +0.4, DV −1.0 for Cg1; and AP +2.7, ML +2.0, DV −5.8 for the AI, with the stereotaxic arm angled 20° toward the right from midline. Coordinates were based on the rat atlas of Paxinos and Watson (2007); all implants were right hemisphere. Half the rats received stimulating electrodes in the Cg1, and the other half received stimulating electrodes in the AI. Each animal was also implanted with recording electrodes for local field potential (LFP) capture; animals with stimulating electrodes in Cg1 had ipsilateral recording electrodes in AI, and vice versa. LFP data are not presented in this report. The electrodes were connected to gold pins inside a McIntyre plug which was cemented to skull screws.

Once surgery was finished, animals were administered a dose of carprofen (5 mg/kg, s.c.), ear bars were removed, and isoflurane was reduced to 0%. After 10 min of breathing oxygen mixture through the nose cone, animals were transferred to a clean, sterile cage and were monitored for 6 h. Animals were then returned to their home cage and closely monitored for 7 days; during this time there was free access to food and water. Animals were returned their pre-surgical food restriction regimen on post-surgical day eight.

### Electrodes and Stimulation

Both stimulating and recording electrodes were made of twisted and PFA-insulated stainless steel wires (diameter 0.005″ bare, 0.008″ coated, SDR Scientific, Australia). The stimulating wires were twisted together, with each tip separated by 0.5 mm; this spacing was selected to limit current spread to a minimum (Bagshaw and Evans, 1976; Tehovnik, 1996). Recording electrodes were arrays constituted of three wires twisted together. The free ends of each electrode were soldered to gold pins and inserted into a McIntyre connector (Molino and McIntyre, 1972).

Electrical stimulation was generated by an isolated, constant current stimulator (Model 4100, A-M Systems, United States). Stimulation trains consisted of biphasic square pulses (100 ms pulse width, 75 μA amplitude); these parameters are based on previous ACC and AI stimulation experiments (Pushparaj et al., 2013; Lim et al., 2015) and preliminary trials in our laboratory. Three frequencies were used separately in different trials: sham stimulation (0 Hz), low frequency stimulation (LFS, 10 Hz), and high frequency stimulation (HFS, 130 Hz), which were delivered uninterrupted throughout the testing session. Stimulation sessions were performed with at least 24 h interval between each session. While in the testing apparatuses, animal movement was tracked via an overhead camera (CV-S3200, JAI, United States) and headstage mounted LEDs.

### Experimental Design

After surgery and recovery, animals were returned to the WLT apparatus for testing, initially to confirm that they recalled the task and there were no post-surgical impairments in performance. The initial cohort of animals (*n* = 4; two with Cg1 stimulation electrodes and two with AI stimulation electrodes) was run through a counterbalanced A-B-C block design, with blocks of sham, LFS, or HFS. Each block contained 3 days of testing, one session per day. A second cohort (*n* = 6; three with Cg1 stimulation electrodes and three with AI stimulation electrodes) was run through a counterbalanced A-B-C-C-B-A block design, with blocks of sham, LFS, or HFS. Each block contained 3 days of testing, one session per day.

For each session, the animal was placed in the WLT arena (with no rope initially present), stimulation was initiated, and the animal was given 2 min of arena exploration. After this baseline period, the 0g rope was fed into the arena and the animals began the WLT. After 10 successful trials (i.e., pulling the rope 30 cm to trigger reward dispensing) on the 0 g rope, a 45 g weight was attached to the rope. The task continued in this progressive manner – where 45 g was added every 10 successful trials – until the animal quit the task or reached the max weight of 225 g. A quit was defined as a 60 s period of being off-task, i.e., no rope pull attempts and no sucrose consumption within 60 s. The weight on which the animal quit the task was deemed the “break weight.” When a quit was determined, the experimenter retracted the rope from the arena and the animal was given a final 2 min of arena exploration. At the end of this 2 min period, two doses of sucrose reward were manually dispensed by the experimenter in order to ascertain if animals quit the task due to satiation. The animal was then returned to its home cage. The floor of the arena was cleaned with a disinfectant liquid (Tego 2001, Hugh Crane, United Kingdom; 1% solution) in between sessions, and illumination of the experimental room was kept to a minimum.

To determine if stimulation of either brain region affected general locomotor behavior, animals also completed a series of open field recording sessions. These sessions were completed in the weeks following completion of the WLT. The circular open field apparatus was 70 cm in diameter, 55 cm high and made of black flexible plastic. The apparatus was placed in the center of the WLT arena, allowing the same recording equipment and the same room parameters (inter-session cleaning, illumination) to be used. Each session was 5 min in duration, one session per day. Animals were given one initial day of habituation (no stimulation), and then stimulation sessions were carried out using the same block format as was used in the WLT, with counterbalanced blocks of sham, LFS, and HFS.

### Perfusion and Histology

**FIGURE 2:**
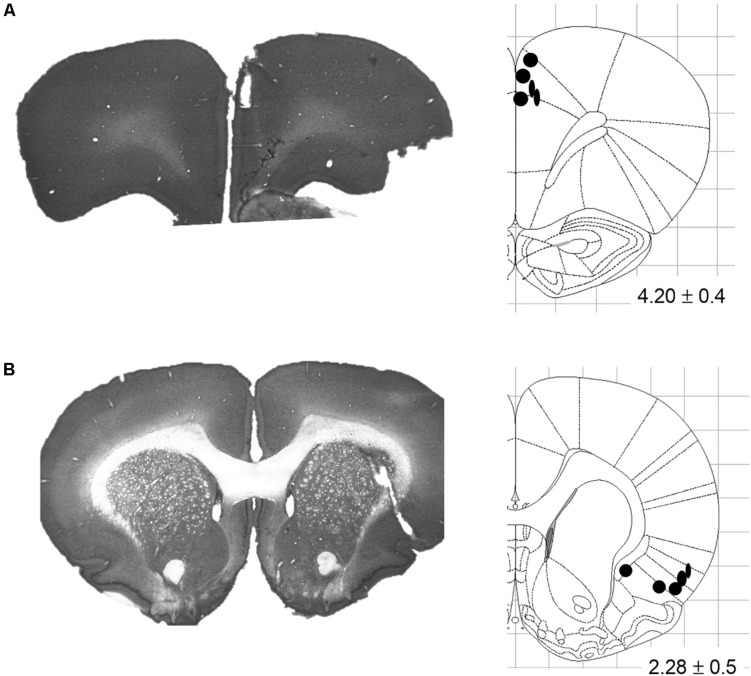
Stimulating electrode placements. **(A)** Representative coronal slice and schematic illustrating terminal tip locations for Cg1-targeted electrodes. **(B)** Representative coronal slice and schematic illustrating terminal tip locations for AI-targeted electrodes. Schematics adapted from the Rat Brain Atlas of Paxinos and Watson (2007); distance from bregma indicated in mm ± anterior-posterior span.

After all experiments were completed, rats were euthanized with isoflurane, and transcardially perfused with saline (0.9%) followed by paraformaldehyde solution (4%) and formalin-sucrose solution (30%). Brains were removed and stored in formalin-sucrose for at least 48 h to allow fixation and to prepare for frozen sectioning. Sectioning was conducted in a microtome-cryostat (Leica CM1860 UV, Germany) at 80 μm thickness, and slices were mounted on clear glass slides. Sections were then stained with thionin and digitally captured, and the locations of the recording and stimulating tips were established according to the rat brain atlas of Paxinos and Watson (2007). All animals had stimulating tips confirmed to be within the borders of the target region (Figure 2).

### Data Analysis

Initial data analyses were carried out using Matlab R2018a and custom Matlab scripts. Video tracking and TTL signals exported from the acquisition system were used to calculate behavioral metrics such as trial duration, attempts-to-success ratio, and time-on-task; all metrics have been detailed previously (Porter and Hillman, 2019; Porter et al., 2020). Collated data were then exported to GraphPad Prism 8.4.3 for statistical analyses and graphing.

To determine if behavioral metrics from the overall session differed between stimulation conditions, normality was first assessed by Shapiro–Wilk. Parametric (one-way ANOVA) or non-parametric (Kruskal–Wallis) tests were then used accordingly to compare conditions, with *post hoc* Dunn’s comparisons made for each stimulation condition versus sham. To determine if intra-session metrics (trial duration and attempts-to-success) differed between stimulation conditions, two-way ANOVA tests were used with factors of Stimulation Condition and Pulling Weight, and *post hoc* Dunnett’s. Asterisks are used throughout to denote significant differences as follows: ^∗^*p* < 0.05, ^∗∗^*p* < 0.01, ^∗∗∗^*p* < 0.001. Since the WLT is designed to allow an animal to quit at any point, and most animals quit before reaching the highest weights of 180 and 225 g, there was a scarcity of trial data at these higher weights. For this reason, we constrained our trial-based comparative analyses to the first four weights – 0, 45, 90, and 135 g – where all ten animals routinely contributed data.

## Results

### Effects of Stimulation on General Motor Behavior

**FIGURE 3:**
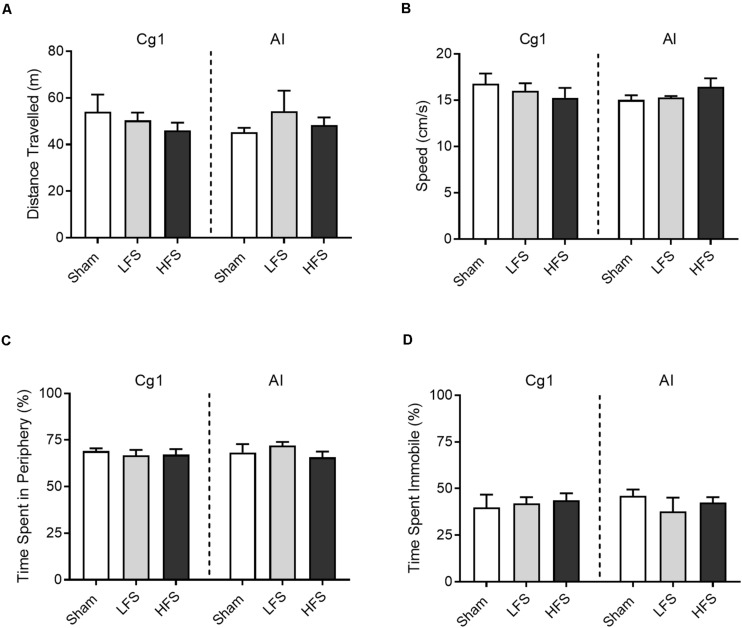
Effects of stimulation on open field behavior. **(A)** Distance traveled. **(B)** Average speed across the 5 min session. **(C)** Time spent in the periphery of the open field, demarcated as within 20 cm of the apparatus’ wall. **(D)** Time spent immobile, defined as moving speed <10 cm/s. Data are shown as mean ± SEM.

No overt motoric effects were observed during delivery of LFS or HFS to either brain region (Figure 3). When tested in a circular open field, stimulation of Cg1 or AI did not affect distance traveled as compared to sham [*Cg1: F*(2,16) = 0.51, *p* = 0.61; *AI: H*(2) = 0.92, *p* = 0.21]. Speed was no different between sham, LFS and HFS conditions [*Cg1*: *F*(2,14) = 0.53, *p* = 0.60; *AI*: *F*(2,8) = 0.97, *p* = 0.42]. Likewise the different stimulation conditions did not differ in thigmotaxis [*Cg1*: *F*(2,16) = 0.26, *p* = 0.77; *AI*: *H*(2) = 1.6, *p* = 0.47] or freezing behavior [*Cg1*: *F*(2,16) = 0.12, *p* = 0.89; *AI*: *H*(2) = 1.05, *p* = 0.63].

### WLT Performance

The WLT has recently been detailed and validated by our lab using male Sprague-Dawley rats (Porter and Hillman, 2019). Here we used a specific version of the WLT – the progressive WLT – to challenge the animals in terms of effortful persistence. Rats initially pull a 0 g rope 30 cm to trigger a sucrose reward, thereby completing one trial. After every 10 successful trials the rope is weighted with an additional 45 g. This is repeated every 10 successful trials until the animal quits, or reaches a maximum pulling weight of 225 g (see sections “Weightlifting Task” and “Experimental Design”).

**FIGURE 4:**
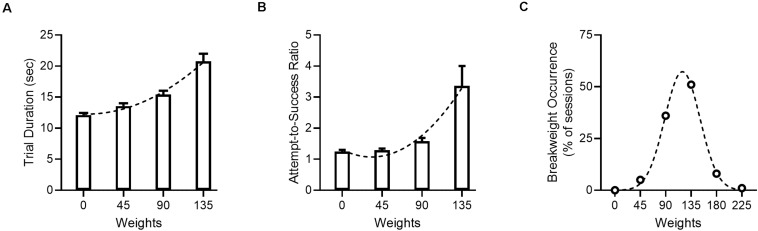
Progressive WLT performance under sham stimulation. **(A)** Trial duration, with one trial being defined as the time from rope pull initiation to reward consumption. Dotted line denotes second order polynomial. **(B)** Attempts-to-success ratio. A ratio of one indicates that a single rope pull attempt was successful and resulted in one reward being dispensed; values >1 indicate that multiple pull attempts were needed to successfully trigger reward. Dotted line denotes second order polynomial. **(C)** Break weight distribution across sham sessions. Open circles denote observed frequencies, dotted line denotes Gaussian fit. Data for panel **(A,B)** are shown as mean ± SEM.

Under sham stimulation conditions (*n* = 43 sessions), animals in this study performed the WLT as expected (Figure 4) and behavioral metrics aligned with metrics observed in previous cohorts (Porter and Hillman, 2019; Porter et al., 2020). As the pulling weight progressively increased, the time to complete a successful pull and earn reward (trial duration) significantly increased [*H*(3) = 50.2, *p* < 0.001], as did the attempts-to-success ratio [*H*(3) = 26.6, *p* < 0.001]. The most common break weight was 135 g, occurring in 51% of sham sessions.

### Effects of Cg1 Stimulation on WLT Performance

**FIGURE 5:**
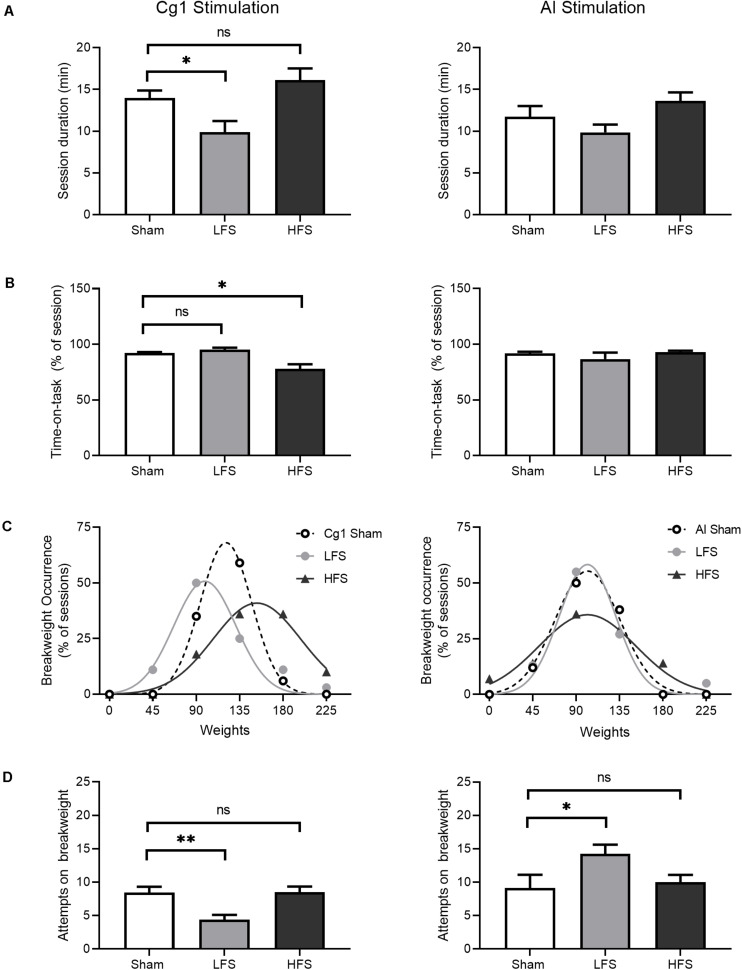
Effects of stimulation on WLT session performance. Cg1 stimulation data are presented on the left, AI stimulation data are presented on the right. **(A)** Session duration. Animals could quit the task at any time point. **(B)** Time-on-task, calculated as the percent of session duration engaged in rope pulling and reward consumption. **(C)** Break weight distribution comparisons between stimulation conditions. Symbols denote observed frequencies, lines denote Gaussian fits. **(D)** Attempts made on the break weight prior to quitting. For all bar graphs, data are shown as mean ± SEM. Asterisks denote significant post hoc comparisons as compared to sham; *p < 0.05, **p < 0.01.

We predicted that low-frequency Cg1 stimulation would increase persistence in the progressive WLT, that is, stimulation would cause animals to work longer and harder in each task session. Session duration did differ between conditions [*H*(2) = 9.7, *p* = 0.008; Figure 5A, left], but stimulation did not result in longer WLT sessions. Rather, *post hoc* comparisons revealed that Cg1 LFS sessions were shorter in duration as compared to sham (*p* = 0.04). Within each session, percentage of time-on-task also differed between conditions [*H*(2) = 19.4, *p* < 0.001; Figure 5B, left], but stimulation did not result in more dedicated time-on-task as predicted. Rather, Cg1 HFS produced a decrease in time-on-task (*p* = 0.03).

Despite this decrease in time-on-task under HFS, HFS sessions were associated with higher break weights (Figure 5C, left). When break weight distributions were fitted with Gaussian curves, bidirectional differences in mean break weight were observed in Cg1 stimulation conditions [*F*(2,9) = 39.9, *p* < 0.001]. Under Cg1 sham conditions, the most common break weight was 135 g (occurring in 59% of Cg1 sham sessions) with a fitted mean ± SD of 121 ± 27 g. Under HFS a rightward shift was observed as compared to sham: the most common HFS break weights were 135 and 180 g (each occurring in 36% of HFS sessions) with a fitted mean of 153 ± 45 g. Under LFS, there was a leftward shift as compared to sham: the most common LFS break weight was 90 g (occurring in 50% of LFS sessions) with a fitted mean of 99 ± 32. Under Cg1 LFS the animals quit the task sooner – as indicated by shorter session durations and lower break weights – and the animals also made fewer attempts on the break weight before electing to quit (Figure 5D, left). When the break weight trials were examined in isolation to determine how many attempts were made before quitting, attempts differed between conditions [*H*(2) = 12.2, *p* = 0.002], with LFS significantly lower than sham (*p* = 0.006).

**FIGURE 6:**
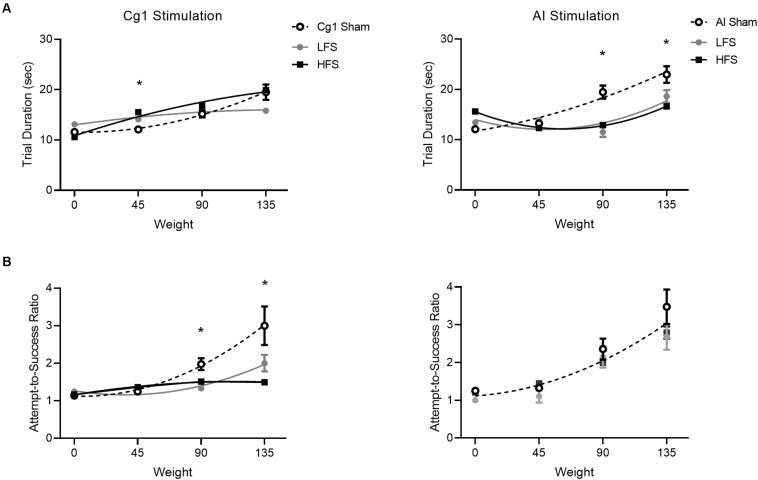
Effect of stimulation on trial performance. Cg1 stimulation data are presented on the left, AI stimulation data are presented on the right. **(A)** Trial duration for stimulations versus sham. **(B)** Attempt-to-success ratios for stimulations versus sham. Data symbols denote mean ± SEM; lines illustrate second order polynomial best fit. For attempts-to-success ratios during insula stimulation there is only one line of best fit illustrated, as all three conditions could be fit with a shared global curve. Asterisks denote significant *post hoc* comparisons where both stimulation conditions (LFS and HFS) differed from sham.

To determine if Cg1 stimulation had more subtle effects on performance within the task itself, trial duration and attempts-to-success ratio were examined across the three stimulation conditions (Figure 6, left). Trial duration exhibited a significant Condition × Weight interaction [*F*(6,396) = 10.3, *p* < 0.001]. The data were best represented by distinct quadratic fits [*F*(6,399) = 10.3, *p* < 0.001]. When attempts-to-success ratios were examined, a significant Condition × Weight interaction was also observed [*F*(6,396) = 5.9, *p* < 0.001]. Again the different stimulation conditions were best represented by distinct quadratic fits [*F*(6,399) = 8.4, *p* < 0.001].

### Effects of AI Stimulation on WLT Performance

We predicted that AI stimulation would reduce effort investment in the WLT, that is, animals would spend more time off-task and/or quit the task sooner. When overall session durations were compared, no effect of AI stimulation was detected [*H*(2) = 5.1, *p* = 0.08; Figure 5A, right]. Within each session, percentage of time-on-task also did not differ between conditions [*H*(2) = 0.39, *p* = 0.82; Figure 5B, right]. When break weight distributions were fitted with Gaussian curves, mean break weights were no different between conditions [*F*(2,9) = 3.2, *p* = 0.08; Figure 5C, right]. When break weight trials were examined in isolation to determine how many attempts were made before quitting, stimulation did have a small, marginally significant effect [*F*(2,42) = 3.1, *p* = 0.055; Figure 5D, right]. Low-frequency stimulation of the AI was associated with an increase in the number of attempts on the break weight as compared to sham (*p* = 0.046).

To determine if AI stimulation had more subtle effects on performance within the task itself, trial duration and attempts-to-success ratio were examined across conditions (Figure 6, right). Trial duration exhibited a significant Condition × Weight interaction [*F*(6,477) = 8.24.1, *p* < 0.001]. The data were best represented by distinct quadratic fits [*F*(6,480) = 8.6, *p* < 0.001]. When attempts-to-success ratios were examined, there was no Condition × Weight interaction [*F*(6,477) = 1.1, *p* = 0.37]. The same curve fit could be applied to all conditions [*F*(6,480) = 1.9, *p* = 0.08].

## Discussion

Given that conjoint activation of the cingulate cortex and insular cortex is observed during effortful decision-making (Engstrom et al., 2014), volitional responding (Medford and Critchley, 2010), and task switching (Menon and Uddin, 2010), we hypothesized that stimulation of these areas in rat would alter performance in an effortful weightlifting task. Despite being used clinically, electrical stimulation *in vivo* is still not well understood in terms of mechanism. Stimulation can cause proximal as well as distal effects and has been linked to neuronal excitation as well as inhibition; stimulation frequency appears to be one important determinant in these differing effects (Bari et al., 2013; Mohan et al., 2020). For this reason we tested two stimulation frequencies – 10 and 130 Hz – frequencies which have been used successfully in prior rat studies (Pushparaj et al., 2013; Rea et al., 2014; Lim et al., 2015; Lindenbach et al., 2019). We initially predicted bidirectional effects based on stimulation site: that Cg1 stimulation would increase effort expenditure and persistence in the task, and that AI stimulation would decrease effort expenditure and prompt earlier quitting.

Contrary to our prediction, low-frequency Cg1 stimulation resulted in shorter task sessions: animals quit the task sooner (lower break weight) and made less attempts on the break weight before quitting. One interpretation of this is that LFS reduced motivation to perform the task, notably as it got more difficult, however performance metrics within the session indicated that there was motivated rope-pulling throughout. At 0 and 45 g LFS was associated with slower trial-by-trial performance (longer trial duration) but the attempts-to-success ratio centered around 1; the latter continued into higher weights indicating that when rats decided to initiate a pull they were generally successful. One interpretation of this is that Cg1 LFS facilitates a “slow and steady” approach, where the time to complete a single trial is slower but the efficiency of the pull is maintained (i.e., an attempt-to-success ratio ∼1), even at higher pulling weights. This differs from sham stimulation conditions, where there is an exponential increase in attempts-to-success ratio as the pulling weight increases. Under sham stimulation in this study, and as observed in non-stimulated animal cohorts in previous studies (Porter and Hillman, 2020; Porter et al., 2020), heavier weights result in more failed attempts – i.e., rope pulls fail to reach the 30 cm mark required to trigger reward. Cg1 LFS appears to exert a subtle change in WLT behavior: the animal experiences fewer fails, but also terminates the task earlier.

High-frequency Cg1 stimulation in our animals was associated with more time spent off-task in each session, however this did not equate to poorer overall performance in the task. Rather, HFS was again associated with a “slow and steady” pulling efficiency but also with a higher break weight. Taken together these data suggest that under Cg1 HFS animals work consistently at the task, even into higher weights, but take frequent small breaks which culminate in more time spent off-task. To our knowledge this is one of the first studies to examine rat Cg1 modulation during an effortful task. Hart et al. (2020) also recently published a study examining Cg1 modulation during an effortful task; they demonstrated that chemogenetic excitation and inhibition of the region reduced lever-pressing in a progressive ratio, choice-based task. Similar to our study, the Hart et al. (2020) findings defy simple interpretation: manipulations assumed to be opposing produced similar behavioral shifts. While puzzling, both studies demonstrate that Cg1 manipulation during a task can shift effort expenditure in subtle ways.

Electrical stimulation of the rat cingulate and deeper vmPFC has been examined in other previous studies that were framed toward investigating anxiety- and depressive-like behaviors. However, results of those studies have been mixed. For example, Lim et al. (2015) compared the effects of 10 and 100 Hz Cg1/vmPFC stimulation in naive Sprague-Dawley males as the animals performed a battery of tests. HFS reduced home cage emergence latency and increased food intake, however null effects were reported in the open field, sucrose intake test and forced swim test. Rea et al. (2014) used 130 Hz vmPFC stimulation in rats from the Flinders Sensitive Line, a genetic animal model of depression, and found that HFS improved sucrose consumption and forced swim performance. Our findings that LFS and HFS of Cg1 produce subtle but significant changes in WLT performance in naïve animals suggests that the WLT may be worth investigating in future rodent studies investigating anxiety- and depressive-like behaviors.

Low-frequency insular stimulation in our study was associated with increased attempts on the break weight and faster performance (shorter trial duration) on higher weights. Faster performance on higher weights was also observed in the HFS condition for AI. In both stimulation conditions, there was no difference in attempts-to-success ratio as compared to sham, and no difference in end break weight or time on-task as compared to sham. Taken together, one interpretation is that AI stimulation (LFS or HFS) increases the speed of the animal’s trial-by-trial task performance in high-effort circumstances, but this does not equate to improved efficiency or improved performance overall (i.e., higher break weight). In our open field assessment HFS in the AI initially appeared to induce a slight increase in speed (Figure 3) however this was not a significant difference compared to sham (*p* = 0.38). Thus the change in speed observed in our animals appears task-specific and not a general change in locomotor activity, a finding that has also been observed from stimulation of the lateral habenula in male Wistar rats (Jakobs et al., 2019).

In a previous insular stimulation study in rat, Pushparaj et al. (2013) demonstrated that 130 Hz HFS decreased nicotine self-administration in fixed-ratio and progressive-ratio operant tasks. These behavioral results mirrored the group’s earlier findings using baclofen inactivation of in the insula (Forget et al., 2010) leading the authors to suggest that HFS is producing a regional inactivation effect. In our study, however, we found no significant effect of AI HFS on progressive-ratio performance overall (i.e., break point) for sucrose reward. Likewise, a prior study using quinolinic acid lesioning of the insula also reported no effect on progressive-ratio responding for normal food pellets (Daniel et al., 2017). Thus it is difficult to interpret just how insula manipulation alters effort expenditure – whether it affects motivation, effort exertion, or more generally the decision-making framework.

Lesioning of the rat anterior insula by various methodologies suggests it may be at the more general level of decision-making framework. Optimal choice selection in a slot machine task is reduced following GABAergic inhibition of the insula (Cocker et al., 2016); decision-making in a rodent version of the Iowa Gambling Task is altered following quinolinic lesioning of the insula (Daniel et al., 2017); and strategy shifting in response to sensory specific satiety is reduced following chemogenetic manipulation of the insula (Parkes et al., 2018). Importantly, Daniel et al. (2017) highlighted the impact of the individual rat’s baseline behavioral preference pre-insular lesion, and this may be worth considering in regional stimulation studies. Daniel et al. (2017) demonstrated that insular inactivation in baseline “good decision makers” caused a shift toward less-optimal exploitation behavior, whereas insular inactivation in baseline “poor decision makers” caused a shift toward more optimal exploitation. Individual variability in gambling decisions likely explains the discrepant results that other groups reported in insular manipulation gambling studies which analyzed group means (Mizoguchi et al., 2015; Pushparaj et al., 2015). Because of the small sample size in the current study (*n* = 10), we were reliant on group means and could not perform robust group splits into baseline “low-effort” and “high-effort” rats, however this is an area ripe for future investigation.

Finally, for future studies of Cg1/AI stimulation, we offer two methodological considerations. In this study we utilized unilateral stimulation; it would be interesting to repeat the WLT using bilateral stimulation to determine if the subtle effects we observed here become more prominent with bilateral stimulation. Indeed many of the studies discussed above utilized bilateral stimulation (Pushparaj et al., 2013; Rea et al., 2014; Lim et al., 2015; Lindenbach et al., 2019). In this study we also utilized continuous stimulation for the duration of the task session, similar to the studies discussed above. One innovative stimulation approach has recently been detailed by Lindenbach et al. (2019). Their study examined 20 Hz stimulation of the ventral subiculum during a progressive-ratio task, but rather than continuous stimulation the researchers applied stimulation only after the first fail (± stimulation at the start of the session). This idea of using stimulation to “boost” performance after a failed attempt lends itself well to the WLT and combined with bilateral stimulation could provide interesting results.

## Data Availability Statement

The raw data supporting the conclusions of this article will be made available by the authors, without undue reservation.

## Ethics Statement

The animal study was reviewed and approved by the University of Otago Animal Ethics Committee, AUP 91/17.

## Author Contributions

KLH and BSP conceived the study. CS ran the experiments. BSP wrote analysis routines. All authors participated in data analysis and manuscript preparation.

## Conflict of Interest

The authors declare that the research was conducted in the absence of any commercial or financial relationships that could be construed as a potential conflict of interest.
